# Cost Modifications during the Early Years of the Use of the National Cardiovascular Data Registry for Quality Improvement

**DOI:** 10.6061/clinics/2020/e1708

**Published:** 2020-08-21

**Authors:** Pedro Gabriel Melo de Barros, John Li, Christine Tremblay, Mariana Yumi Okada, Henry Sznejder, Valter Furlan, Rafael Vasconcellos

**Affiliations:** I Hospital Samaritano Paulista, Sao Paulo, SP, BR.; II Optum Health LLC, Eden Prairie, Minnesota, US.; III UnitedHealth Group Brazil, Rio de Janeiro, RJ, BR.

**Keywords:** Cardiovascular Disease, Quality Improvement, Appropriate Health Care

## Abstract

**OBJECTIVES:**

Quality improvement (QI) initiatives based on data from international registries have been reported previously; however, there is a lack of information on the impact on the costs of medical care associated with the use of these tools.

**METHODS:**

Patients admitted due to myocardial infarction (MI), included in the ACTION Registry® and CathPCI Registry®, in a private Brazilian hospital (i.e., the reference hospital) were analyzed. The costs of care of these patients were compared to the costs of MI admissions in nine similar hospitals not included in the same QI program. Regression models were used to analyze the cost change over time between the two groups of hospitals. Readmission rates were compared using logistic regression, adjusting for the same variables as in the cost model.

**RESULTS:**

Overall, the annual medical cost inflation in Brazil was higher than the annual cost trend in the reference hospital during the period of analysis. Moreover, the annual in-hospital costs indicate that the reference hospital has a statistically significant 6% lower cost trend for patients with acute MI, compared to patients with the same diagnostic code in the comparison hospitals group, in an adjusted analysis (*p*-value=0.041). Using multivariable analysis, the readmission rates were also found to be significantly lower in the reference hospital than in the comparison hospitals, with an odds ratio of 0.68 (*p*-value=0.042).

**CONCLUSION:**

The use of the NCDR® as a benchmark to guide QI programs outside the United States was associated with the positive impact of bending the cost curve to below that of national medical inflation and the comparison hospitals’ costs, with a lower incidence of hospital readmission.

## INTRODUCTION

Cardiovascular disease is the most common cause of death worldwide; among all its different types of events, acute coronary syndromes (ACS) account for half of these deaths (1,2). Beyond their clinical relevance, ACS represent a significant economic burden globally. Previous analysis of direct medical expenditure on ACS patients in the United States has revealed a cost of over US$150 billion annually; approximately 70% of this cost is related to hospital admission and readmission (2-4). Additionally, an analysis of health care costs in Brazil in 2015 reveals that treatment/care of myocardial infarction (MI) is the costliest among cardiovascular diseases, reinforcing the priority of addressing this condition through comprehensive initiatives (5).

Adherence to guideline-based recommendations on clinical practice that can be measured by large registries is associated with improvement in clinical outcomes of patients hospitalized due to ACS (6-8). These registries, commonly accessed in the United States, have been successfully utilized for continuous quality improvement (QI) programs in other countries as well (9,10). The report of an innovative experience using the national cardiovascular data registry (NCDR®) database in an international site reveals an improvement in the overall performance of quality indicators measured in patients with MI, from 95.0% to 99.6% (*p*-value for trend <0.001) (10). These findings reinforce the promising potential of large global databases to be a robust source of information guiding QI initiatives in cardiology, even in international sites. Nevertheless, there is a lack of information on the impact on the costs of care related to the improvement in quality performance guided by the NCDR® data. The present study aims to analyze the change in the costs of health care delivered by the reference hospital during the early years of the use of the NCDR® data reports as a guide to develop and monitor QI programs. The trends of costs and readmission rates have been compared to those of patients with MI admitted in the comparison hospitals group located in the same geographic region.

## METHODS

### Study design

This was a multicenter, retrospective analysis of patients admitted with a diagnosis of MI in 10 Brazilian hospitals.

### Study objectives

Primary objectives:

– To evaluate the trend of in-hospital costs related to MI admissions during the years of QI using the NCDR® database, in comparison to medical inflation (11)
– To evaluate the trend of in-hospital costs related to MI admissions during the years of QI using the NCDR® database, in comparison to similar hospitals located in the same region

Secondary objectives:

– To evaluate the risk of readmission of MI patients, as per the hospital (i.e., the reference hospital or comparison hospitals group)
– To evaluate the costs of readmission of MI patients
– To evaluate the length of stay (LOS) of MI patients, as per the hospital (i.e., the reference hospital or comparison hospitals group)
– To evaluate in-hospital costs according to the procedure performed during hospitalization of MI patients

### Study participants

Patients admitted due to MI from January 2013 to December 2016 in 10 different hospitals were included in the present study. The reference hospital was compared to nine similar hospitals located in the same city (i.e., São Paulo, Brazil). The reference hospital in the present study was a private general hospital with international quality accreditation, and was part of an international center of excellence (ICOE) quality program by the American College of Cardiology that included the use of the NCDR®. The results related to QI were published previously (10).

The following criteria were used to select the hospitals to be compared with the reference hospital: **1.** Private hospital in the same city (i.e., São Paulo, Brazil), **2.** international quality accreditation, and **3**. not a participant in the ICOE quality program by the American College of Cardiology.

Admissions to these hospitals were included in the analysis if they met the following criteria: **1.** Hospital admission due to MI, and **2.** similar procedure type during admission (i.e., coronary angiography, percutaneous coronary intervention (PCI), or coronary artery bypass graft (CABG) surgery).

Anonymized records containing data such as costs, diagnostic codes, and procedure codes from the reference hospital and the comparison hospitals were sourced from the same Brazilian private health insurance payer. There could have been more than one admission for a patient during the period. Diagnosis and procedure codes on admits from these hospitals were used to select similar admissions between January 2013 and December 2016. This period was selected for the study due to data availability from the hospitals included in the analysis.

### Evaluation of costs and readmissions

The costs were assessed from the perspective of the health insurance company. The same methods were used to calculate costs (i.e., both admission and readmission costs) for all hospitals. Administrative claims were used for this analysis; thus, costs included everything billed to the insurer for each admission. Claims were consolidated using the authorization number that was required for payment on all claims for the same admission, regardless of source. The analysis compared the year-over-year cost variance between MI patients at the reference hospital and those at the comparison hospitals in the same geographic region, using anonymized claims data from the same Brazilian private insurer. Regression methods were used to adjust for known variables. Additionally, readmission rates based on the health insurance claims for those admissions were compared between the two groups.

### Statistical analyses

Categorical variables were reported by the absolute and relative frequencies, and quantitative variables were described by the mean and standard deviation. Regression models were used to analyze the cost differences between the two groups of hospitals. A gamma log model was used with adjustments for the patient’s treatment type, sex, age, and benefit plan. Additional clinical information was not available for the reference hospitals; hence, further adjustment was not feasible. The main variable of interest was the interaction between the hospital group and year to determine the cost change over time. Readmission rates were compared using logistic regression, adjusting for the same variables as in the cost model. Cost outliers, defined as the total cost above the 99^th^ percentile and below the 1^st^ percentile, were excluded from the analysis.

Odds ratios (ORs) were reported with the corresponding 95% confidence interval and *p*-value. While the *p*-values were two-tailed, those below 0.05 were considered statistically significant. The data were statistically analyzed with SAS® 9.4 (SAS Institute Inc.).

## RESULTS

**Figure 1:**
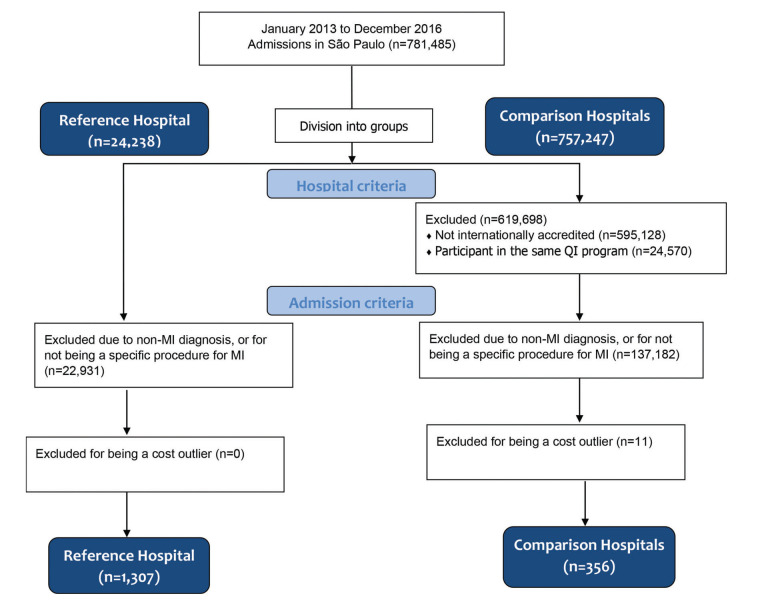
Consort diagram.

A total of 1,663 admissions of 1,610 patients from 10 hospitals in São Paulo, Brazil between January 2013 and December 2016 were included (Figure 1). Since the information from the comparison hospitals was sourced from the administrative database, the characteristics of the population analyzed included the age, sex, and type of procedure. The mean age was 59.9±12 in the reference hospital, and 66.3±15 in the comparison hospitals group (*p*-value<0.01). There were more males in both groups: 69% (n=900/1307) in the reference hospital, and 65% (n=231/356) in the comparison hospitals group (*p*-value=0.15). Additionally, the percentage of PCIs was not statistically different for the two groups: 58% (n=760/1307) and 54% (n=191/356) in the reference hospital and comparison hospitals group, respectively (*p*-value=0.13).

### Trend of in-hospital costs related to MI admissions during the years of QI using the NCDR® database, in comparison to medical inflation

Overall, the annual medical cost inflation in Brazil was higher than the annual cost trend in the reference hospital, during the same period as this study (Table 1).

### Trend of in-hospital costs related to MI admissions during the years of QI using the NCDR® database, in comparison to similar hospitals’ costs

**Figure 2:**
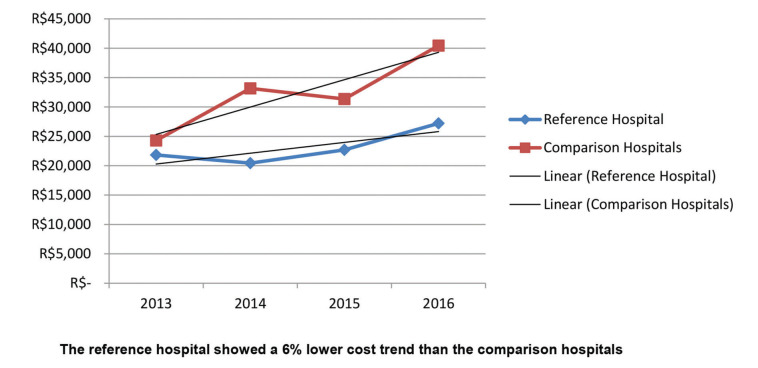
Adjusted cost analysis of acute MI admissions in the reference hospital *versus* comparison hospitals.

**Figure 3:**
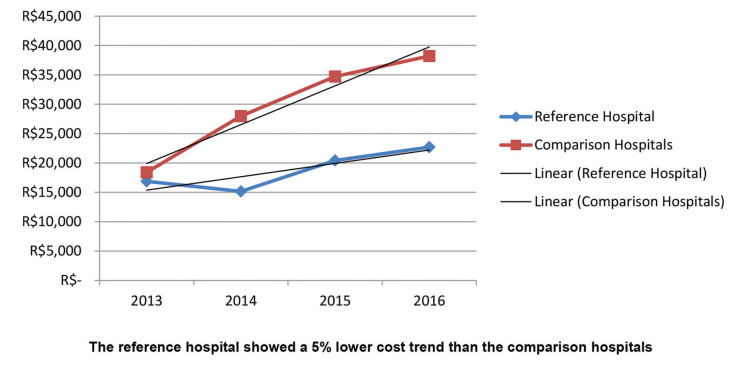
Adjusted cost analysis of the PCI subgroup in the reference hospital *versus* comparison hospitals.

**Figure 4:**
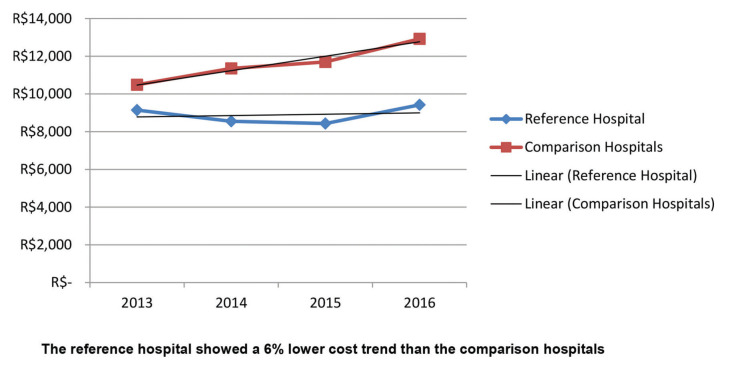
Adjusted cost analysis of the medical treatment subgroup in the reference hospital *versus* comparison hospitals.

The results of the study showed that the adjusted costs were similar for the reference hospital and comparison hospitals in 2013; however, the curves separated over the years (Figure 2). The trend of increase in the costs for patients with Acute MI in the reference hospital was statistically significantly lower when compared to similar patients in the comparison hospitals. Although not significant, the trend was similar when patients were stratified by PCI and medical treatment (Figures 3 and 4). The number of CABG surgery patients was too low to perform a specific analysis.

### Readmission rates and costs

The observed readmission rate in the reference hospital was 11.5% *versus* 14.5% in the comparison hospitals group, for all MI admissions in the study period; using logistic regression adjusting for age, sex, benefit plan, and treatment type, the readmission rate was significantly lower in the reference hospital than in the comparison hospitals, with an OR of 0.68 (*p*-value=0.042). While the cost savings from avoided readmissions were not factored into the overall cost comparisons, the average readmission cost was R$ 23,217.

### Length of stay

**Figure 5:**
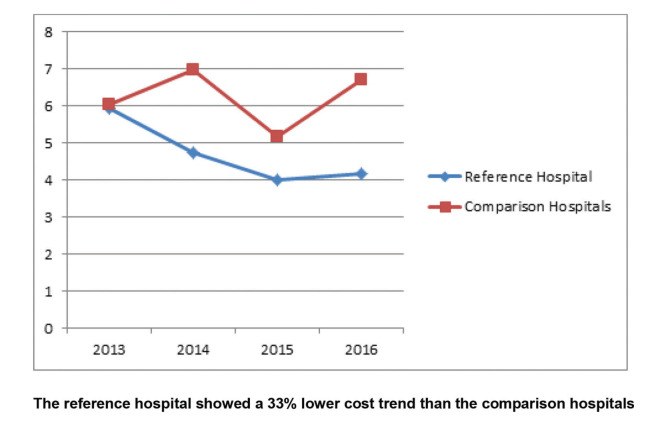
Average LOS by year and group.

With regard to the hospital admissions for MI during the period of analysis, the reference hospital showed a significantly lower average LOS than the comparison hospitals, of 4.6±3.6 days *versus* 6.2±4.8 days, respectively (*p*-value<0.001). Additionally, the change in LOS at the reference hospital over time, demonstrated a decreasing trend (*p*-value for trend <0.001) that was not apparent in the comparison hospitals (Figure 5).

## DISCUSSION

The present study evaluated the costs of in-hospital care of MI patients during the period of QI using the NCDR® as a tool for guiding the QI initiatives (10). The analysis showed that the adjusted costs of the reference hospital and comparison hospitals were similar in the first year of analysis; however, the improvement in quality indicators related to cardiovascular care during the subsequent years was associated with an annual change in costs lower than the national medical inflation, and 6% lower than the costs of other hospitals from the same geographic region with the same international quality accreditation. In addition to the lower cost of health care during hospitalization, the reference hospital population of MI patients had a lower incidence of readmission.

The relationship between quality and costs in health care is generally described as the core of value-based medicine or value-based health care (12,13). This value is directly related to the quality of care and to patient outcomes, and inversely related to the costs of health care (12-14). The addition of patient experience to this equation leads to the triple aim that represents the target of various health care systems currently (14). Offering an appropriate treatment in the scenario of previously high quality of care is generally not associated with an increase in health care costs; consequently, the value of the medical care increases in this scenario (12-17). Nevertheless, despite this theoretical relationship, it is important to include cost analysis in all QI initiatives, since the addition of this information is essential for identifying the indeed value of each initiative, thereby providing more information for decisions in health care policies. There is a lack of this type of information on QI initiatives, especially in regions such as Latin America (14). The present study provides additional information not only on the value of QI benchmarks such as the NCDR® in countries outside the United States, but also on the potential value of global QI initiatives.

In addition to the aim of improving the quality of care, analyzing the effect of the quality change on the costs of health care is critical because this relationship may vary according to the scenario (15-18). In a scenario of low technology and limited human resources QI requires financial investment, as observed in the United States in the second half of the twentieth century (18). However, in a scenario of clinical practice that involves frequent over-diagnosis, overtreatment, and waste of resources, the improvement in appropriateness of procedures and in the performance of quality indicators may reduce health care costs or, at least, reduce the magnitude of cost enhancement (14-20). Currently, this second scenario occurs in contemporary practice in various countries, including a large number of hospitals and medical facilities worldwide, especially in high-income countries (15-23). With regard to the medical practice of middle- and low-income countries, both scenarios may be identified (24,25). Thus, the improvement in quality might relate to the increase or decrease in costs, depending on the hospital or medical system where this process occurs. Nevertheless, even in the context of increasing costs, this enhancement in both metrics (i.e., quality and costs) should be measured in order to provide complete information for decisions in value-based health care. In the present study, the lower magnitude of cost modifications, below medical cost inflation, was associated with a reduction in the LOS. This reduction could be the consequence of more efficient processes of care, a lower rate of clinical complications, or both; this was the probable explanation in the present study, since not only the LOS, but also the rate of complications among MI patients reduced during the period of analysis (10).

Finally, an analysis of costs that addressed only the in-hospital period could have been flawed if these lower in-hospital costs were associated with higher readmissions. Thus, the lower costs identified in the reference hospital could have been related to higher readmissions. Nevertheless, in the present study, despite the lower costs found compared to medical inflation and the comparison hospitals’ costs, in a multivariable analysis, a 32% lower risk of readmission in the reference hospital was observed. This result reinforced that patient outcomes were not driven primarily by the costs of health care; the quality of care needed to be the primary goal. Thus, if the hospitals started focusing on quality and appropriateness, the patients would have had better outcomes, and probably a better experience, and the costs would have been more controlled, thus maximizing the value of health care and fulfilling the triple aim objective (12-14).

### Study limitations

Since this was not a randomized study, some patient characteristics were different between the groups, as observed with regard to mean age. In order to minimize this issue, the present research included hospitals from the same region only, with similar characteristics. The analysis was adjusted to known variables such as the treatment type, sex, age, and benefit plan, and the cost modifications were assessed in the same hospital over the years. Furthermore, the information from the comparison hospitals was limited to administrative claims data, and we did not have access to the internal QI initiatives of these hospitals. Thus, a direct comparison of the differences in the quality of care was not possible. However, the main objective of the study was to evaluate if the improvement in the quality of care observed in the reference hospital (10) affected the costs of care in relation to medical cost inflation and the cost variance in similar hospitals. Finally, the present study did not include the costs related to purchasing the NCDR® database, tools for data extraction, and hospital staff deployed for data management. These costs should have been taken into account when deciding whether to purchase a QI tool. Nevertheless, the main objective of this study was to evaluate the relationship between QI and costs that could be applied to the different tools supporting improvement in clinical practice effectively.

## CONCLUSION

The use of the NCDR® as a benchmark to guide QI programs outside the United States was associated not only with an improvement in quality indicators, as published previously, but also with the impact of bending the cost curve to below that of national medical cost inflation and the comparison hospitals’ costs. Additionally, the period of QI and lower in-hospital costs of health care for MI patients in the reference hospital was associated with a lower LOS and readmission rates than those observed in similar patients from the comparison hospitals group in the same period. The present study reinforced the need to focus on the impact not only on the quality, but also on costs, to identify the interventions with the best value in current medical practice.

## AUTHOR CONTRIBUTIONS

Barros PGM conceived, designed, and supervised the study, interpreted the data, and drafted and revised the manuscript. Li J and Tremblay C analyzed the data and revised the manuscript. Okada MY, Sznejder H and Furlan V interpreted the data and revised the manuscript. Vasconcellos R designed and supervised the study, interpreted the data, and revised the manuscript. All authors read and approved the final version of the manuscript.

## Figures and Tables

**Table 1:** Comparison of annual medical cost trend with the Brazilian medical inflation index.

Year	Reference Hospital Annual Cost Modification (%)	Brazil Annual Cost Increase – Medical Inflation (%)
2014	-7%	16%
2015	16%	19%
2016	10%	20%
